# Localization and gene expression of steroid sulfatase by RT-PCR in cumulus cells and relationship to serum FSH levels observed during in vitro fertilization

**DOI:** 10.1186/1743-1050-2-6

**Published:** 2005-04-11

**Authors:** Yukiko Otsuka, Atsushi Yanaihara, Shinji Iwasaki, Junichi Hasegawa, Takumi Yanaihara, Takashi Okai

**Affiliations:** 1 Department of Obstetrics and Gynecology Showa University School of Medicine 1-5-8, Hatanodai, Shinagawa-ku, Tokyo, Japan

## Abstract

**Background:**

The purpose of this study was to localize the expression of steroid sulfatase (STS) in cumulus cells and to determine the relationship between STS mRNA expression and the serum levels of follicle-stimulating hormone (FSH), luteinizing hormone (LH), estradiol and progesterone.

**Methods:**

The subject group included 49 women (29 to 44 years old) for whom in vitro fertilization treatment was indicated. All subjects gave informed consent. One hundred fourteen samples of cumulus-oocyte complex (COC) were obtained under microscopic observation. Part of the COC was stained by STS antibody. RNA was extracted by phenol-chloroform method and real-time PCR was performed. Serum of each patient was collected and was measured by ELISA.

**Results:**

Some of the cumulus samples were stained by STS antibody. The expression of STS mRNA in all samples was confirmed by quantitative RT-PCR. Although there was no significant correlation between the level of STS mRNA and the serum levels of estradiol, progesterone and LH, there was a statistically significant negative correlation between the level of STS mRNA expression and the serum level of FSH (n = 105, p = 0.018, r = -0.22).

**Conclusion:**

These results have demonstrated for the first time the expression of STS in cumulus cells by immunohistological stainings and real-time RT-PCR. STS expression in cumulus cells may be related to the control of the local steroidal environment in the oocyte. Serum FSH may control STS mRNA expression from the results of RT-PCR, although the correlation was low.

## Introduction

It has become clear that the interaction between the oocyte and granulosa cells is required for the growth of the oocyte. Granulosa cells are influenced by gonadotropin in the blood and are engaged in hormone regulation in the ovary. As follicles grow and an antrum is formed, granulosa cells separate into two anatomically and functionally distinct sub-types: the cumulus granulosa cells, those surrounding and in intimate metabolic contact with the oocyte; and the mural granulosa cells (MGC), the cells lining the follicle wall forming a stratified epithelium with the basal lamina. Oocyte-regulated pathway of granulosa cell differentiation, through the secretion of paracrine growth factors, is towards the cumulus cell phenotype. Cumulus cells display functional characteristics that are markedly distinct from MGC; they have a high rate of proliferation, very low LH receptor expression compared to MGC, and posses the capacity to secrete hyaluronic acid and undergo mucification/expansion which MGC do not [1-4].

The highly specialized cumulus cells have trans-zonal cytoplasmic processes, which penetrate through the zona pellucida and abut the oocyte membrane [5], forming the cumulus-oocyte complex (COC). Gap junctions at the ends of these processes allow the transfer of small molecular weight molecules between oocyte and cumulus cell, and also between cumulus cells, whereas larger molecules are transported by receptor-mediated endocytosis. This mode of communication is essential for development and fertility [6-8], and is thought to play a key role in disseminating local and endocrine signals to the oocyte via the cumulus cells.

Recently, the COC was suggested to be involved in steroidogenesis [9,10]. Follicular maturation and development are complex processes influenced by both intra- and extra-ovarian events that lead to successful ovulation. It is possible that steroidogenesis in the COC contributes to the quality of the embryo.

The purpose of this study is to localize the expression of steroid sulfatase (STS) in the COC and to examine the relationships between the STS expression level and the serum levels of follicle-stimulating hormone (FSH), luteinizing hormone (LH), estradiol and progesterone.

## Materials and methods

### Patients

The subject group consisted of 49 women (29 to 44 years old) for whom in vitro fertilization (IVF) treatment was indicated. The women were patients at Showa University Hospital. Informed consent was obtained from all patients. 114 samples of cumulus cells were obtained under microscopic observation. 9 samples of cumulus cells from 3 women were used for the immunohistochemistry, while independently, 105 samples from 49 women were used for RT-PCR.

Indications for the IVF procedure in these women included: tubal factor infertility (11 cases, 32 samples); male infertility (10 cases, 30 samples); and unexplained infertility (28 cases, 89 samples).

### Ovarian stimulation

The patients received ovarian stimulation therapy with clomiphene citrate (CC, Clomid; Merrell, Cincinnati, OH) + follicle-stimulating hormone (FSH; Fertinorm-P; Serono, SA, Madrid, Spain) or human menopausal gonadotropin (HMG; Humegon; Organon, Oss, The Netherlands). The LH surge was induced by 5,000 IU of human chorionic gonadotropin (hCG; Gonatropin; Teikokuzouki KK, Tokyo, Japan) when at least two follicles reached a diameter of 17 mm, as determined by vaginal ultrasound. Oocytes were retrieved under ultrasonographic guidance 34 to 35 h after the administration of hCG.

Oocytes were separated from follicular fluid and flush solution. Then, cumulus cells were separated from oocytes, other cellular debris and blood cells under microscopic observation.

Cumulus cells were obtained, and immunohistochemical staining was performed using anti-human STS polyclonal antibody. The phenol-chloroform method was used to extract mRNA from cumulus cells. The expression of STS mRNA was examined by quantitative RT-PCR using primers specific for STS (as described below).

### Immunohistochemical staining for STS in cumulus cells

Tissue specimens of 9 samples of cumulus cells were fixed in 10% neutral-buffered formalin for 24 to 72 hours at room temperature, dehydrated through a graded alcohol series, cleared in xylene and embedded in paraffin. Serial sections (3-μm thick) were cut and stained with anti-human STS polyclonal antibody using the ABC method [11]. This antibody was confirmed the cross reactivity by western blott method.

Negative controls for the immunostaining were carried out with mouse serum instead of primary antibody.

### RNA extraction, reverse transcription and real-time PCR

Cumulus cells obtained for real-time RT-PCR were put into each microtubes, and freeze preservation at -80 degrees was carried out before RNA extraction. Total RNA was extracted from the cells using the phenol-chloroform method. Extracted total RNAs were diluted with 20 μl DEPC water in the absence of Rnase. 5 μl of RNA solutions were used for reverse transcription (RT) using oligo (dT) primers and a TaKaRa RNA PCR Kit (AMV) Ver 2.1 (TAKARA BIO INC, Shiga, Japan) according to the manufacturer's instructions.

TaqMan Universal PCR MasterMix and Assays-on-Demand Gene Expression probes (Applied Biosystems, Foster City, CA, USA) were used for the PCR step. Amplification and detection were performed using the ABI PRISM 7700 Sequence Detection System (Applied Biosystems) with the following profile : 1 cycle at 94 degrees for 10 min, and 40 cycle each at 95 degrees for 15 sec and 60 degrees for 1 min.

The placental RNA with which dilution magnifications differ (×1, ×10, ×10^2^, ×10^3 ^and ×10^4^) were used and each quantity value were set as ×10^4^, ×10^3^, ×10^2^, ×10, ×1, to make a series of standards curve. The threshold cycle (Ct), which was defined as the cycle at a significant value, was given as the mean value. The relative expression of each mRNA was calculated by the comparative Ct method, using ΔCt (the value obtained by subtracting the Ct value of placental STS and GAPDH mRNA from the Ct value of each mRNAof cumulus cells). Specifically, the quantity of target mRNA relative to placental mRNA was expressed as 2^-(ΔCt)^.

Then, we calculated the ratio of the quantity of STS mRNA to that of GAPDH mRNA in each samples, and used the ratio for analysis as the expression values of STS mRNA of cumulus cells [12].

### Statistical analysis

Pearson correlation analysis was done. P value less than 0.05 was considered statistically significant.

### Serum analysis

The levels of gonadotropins (FSH and LH) and steroids (estradiol and progesterone) in the serum from all 49 womens were determined by a commercial enzyme-linked immunosorbent assay kit (AIA-600II, TOSOH, Tokyo, Japan). The sensitivity, intra-assay coefficient of variation (CV) (n = 1 0) and inter-assay CV(n = 20) were as follows: FSH: 0.07 mIU/ml, 1.6–2.3%, 4.0–4.9%; LH: 0.08 mIU/ml, 1.8–2.3%, 1.9–2.3%; estradiol: 13 pg/ml, 2.4–5.1%, 3.3–7.7%; progesterone: 0.02 ng/ml, 4.4–9.0%, 5.8–10.1%.

## Results

**Figure 1:**
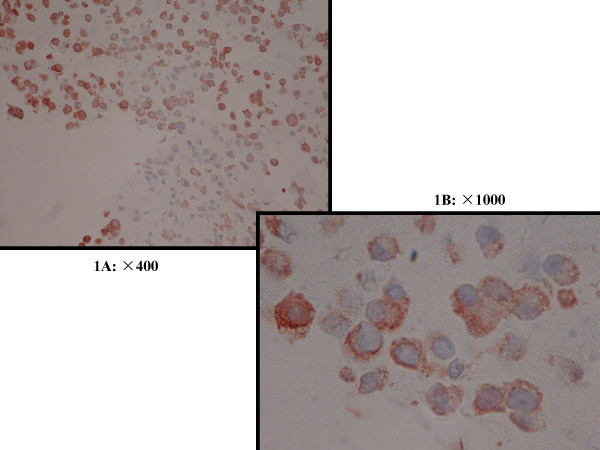
Immunohistochemical staining for STS of cumulus cells1A: ×400, 1B: ×1000 STS expression was present in all 9 samples of human cumulus cells that were immunohistochemically stained. The cytoplasm of the cumulus cells was stained by anti-human STS polyclonal antibody.

STS expression was present in all 9 samples of human cumulus cells that were immunohistochemically stained. The cytoplasm of the cumulus cells was stained by anti-human STS polyclonal antibody, as shown in Fig 1A and 1B.

**Figure 2:**
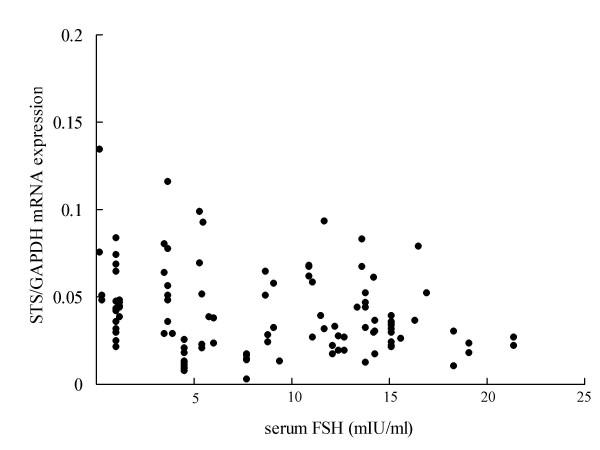
Relationship between expressions of STS mRNA and FSH concentrations of serum. There was a statistically significant negative correlation between the serum level of FSH and STS mRNA expression(n = 105, p = 0.018, r = -0.22)

There was a statistically significant negative correlation between the serum level of FSH and STS mRNA expression (n = 112, p = 0.018, r = -0.22) (Fig 2A). On the other hand, there were no significant correlations between STS mRNA and the serum levels of estradiol (Fig 2B), progesterone and LH.

## Discussion

Follicular maturation and development are complex processes influenced by both intra- and extra-ovarian events that lead to successful ovulation. The follicular fluid concentration of progesterone in mature COC was significantly higher than that in the immature stage [13]. It has been suggested that steroid concentrations in the follicular fluid are associated with oocyte maturation and embryo quality [14-16].

Vanderhyden et al. [10] raised the possibility that mouse oocyte secrete a factor(s) that inhibits progesterone and stimulates estradiol production by cumulus cells by culturing mouse COC and the complex which oocyte was removed microsurgically. The involvement of the oocyte in the regulation of progesterone production suggested a potentially important role for the oocyte in the prevention of premature luteinization of the follicle. Although growth differentiation factor-9 (GDF-9), GDF-9B and/or bone morphogenetic protein-6 (BMP-6) are likely candidate molecules, the identities of these oocyte-secreted growth factors regulating key ovarian functions (prevention of premature luteinization, enabling cumulus cell expansion, extracellular matrix stability, thus facilitating ovulation) remain unknown [4].

Direct production of gonadal steroids from sulfated androgens may be an important alternative or complementary pathway for ovarian steroidogenesis. The conversion of sulfated adrenal androgens, present in serum at micromolar concentrations in adult women, into unconjugated androgens or estrogens requires STS activity.

Although STS activity has been observed in granulosa cells from rats and humans [17,18], the existence of STS in the COC of any species has not been reported. This is the first study to use immunohistochemistry and quantitative RT-PCR to demonstrate that STS is localized and expressed in human COC.

Kosmath et al. reported that the proofs of aryl sulphatase activity gave positive results in all cell types of the atretic follicle. A very strong reaction was observed in the granulosa cells and the migrating cells of the atretic follicle. They raised the possibility that sulfatase might increase during atresia [19]. In this study, we performed the quantitative RT-PCR of STS mRNA in cumulus cells, however, we didn't analyze between the quantity of STS mRNA expression and the maturation of oocyte. We consider it as the future subject to examine the relationship between them.

Schoenfelder et al. [9] reported that bovine COCs were able to secrete several steroid hormones during in vitro maturation (IVM) without the support of follicular granulosa or theca cells and that the COCs possessed some important steroidogenic enzymes, such as 3β hydroxysteroid dehydrogenase (3βHSD) and aromatase. Their results indicated that COCs were able to modulate their steroidogenic environment in vitro. And it is possible that steroidogenesis may include STS. Although rapid morphological and steroidogenic changes are described for granulosa cells [20], in culture granulosa cells responded well to LH and FSH by increased estradiol production besides almost unchanged testosterone and progesterone secretion. Supporting such unaffected progesterone levels, 3βHSD mRNA expression showed no significant changes during gonadotropin treatment. In contrast, the initial increase in aromatase mRNA that was caused by either FSH or LH led to delayed secretion of estradiol, whereas co-treatment induced just slightly increased estradiol levels [9]. These results may indicate an estrus cycle-dependent reactivity of granulosa cells to different gonadotropin ratios, which exist in distinct but changing ratios in vivo. It is likely that gonadotropins control steroidal enzymes, and FSH may control STS expression in cumulus cells.

J Bonser et al. [21,22] reported that there was STS activity and the ability to utilize dehydroepiandrosterone sulfate (DHEAS) as a precursor for estradiol production in human granulosa cells, and that LH and estrone 3-O-sulfamate inhibited this activity. But, they were unable to find any consistent effects of FSH on the conversion of DHEAS to other steroids [21,22].

It was reported that interleukin (IL)-1β in human endometrium suppressed STS mRNA and STS activity in stromal cell culture [23]. Another report indicated that IL-1β in rat granulosa cells mediated the inhibition of gonadotropin-stimulated steroidogenesis by modulating 20α-hydroxysteroid dehydrogenase. The report suggested a role for IL-1β in mediating the observed decline of these bioactive hormones [24]. In humans, FSH stimulated IL-1β secretion by mononuclear cells isolated from the peripheral blood of women in the follicular phase [25]. Previous studies may support the present results; that is, serum FSH levels might control and suppress the STS mRNA expression in cumulus cells through cytokines such as IL-1β.

There might be higher relationship to intrafollicular rather than serum factors. Originally, we should analyze with the hormone levels in follicular fluid, however, we did not perform it in this study. Additionally, because the number of follicles grown by COH in each patients were different and multiple, the local hormonal environment in each follicles would not be reflected in the serum hormonal levels of estradiol and progesterone. Take these speculations into consideration, there may be some correlations with the other hormones of which no correlation was seen between expression of STS mRNA in this study.

In the conclusion, these results have demonstrated for the first time the expression of STS in cumulus cells by immunohistological stainings and real-time RT-PCR. STS expression in cumulus cells may be related to the control of the local steroidal environment in the oocyte. Although serum FSH may control STS mRNA expression from the results of RT-PCR, we think further study is needed on this topic, because the correlation was low. After that, additional research will be also needed to analyze with follicular fluid, to identify the responsible mechanism and to determine the relationship between STS expression in cumulus cells, the maturation of oocyte and quality of the embryo.

**Figure 3:**
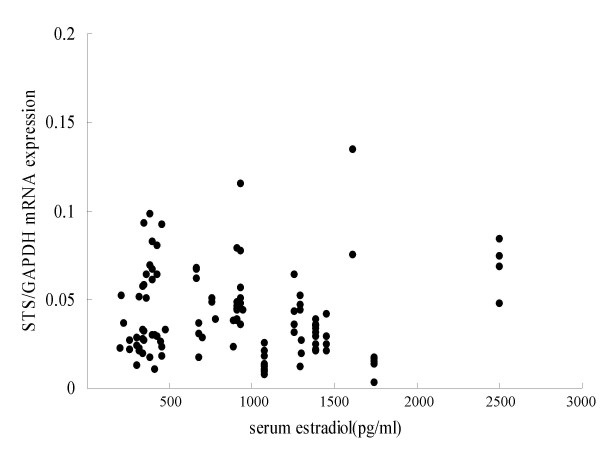
Relationship between expressions of STS mRNA and estradiol concentrations of serum. There were no significant correlations between STS mRNA and the serum levels of estradiol (n = 105, NS), progesterone (n = 105, NS)(data not shown) and LH (n = 105, NS) (data not shown).
